# The immunotherapy breakthroughs in cervical cancer: Focus on potential biomarkers and further therapy advances

**DOI:** 10.17305/bb.2025.12597

**Published:** 2025-05-22

**Authors:** Maja Pezer Naletilić, Krešimir Tomić, Kristina Katić, Zoran Gatalica, Gordan Srkalović, Eduard Vrdoljak, Semir Vranić

**Affiliations:** 1 Department of Oncology, University Hospital Center Mostar, Mostar, Bosnia and Herzegovina; 2 Department of Gynecologic Oncology, University Hospital Center Zagreb, Zagreb, Croatia; 3 Reference Medicine, Phoenix, Arizona, United States of America; 4 University of Michigan Health-Sparrow Herbert-Herman Cancer Center, Lansing, Michigan, United States of America; 5 Department of Oncology, University Hospital Center Split, Split, Croatia; 6 College of Medicine, QU Health, Qatar University, Doha, Qatar

**Keywords:** Cervical cancer, CC, immunotherapy, immune checkpoint inhibitors, ICIs, biomarkers

## Abstract

Despite the well-established role of human papillomavirus (HPV) as the primary cause of cervical cancer (CC) and the existence of an effective HPV vaccine, over half a million women are diagnosed with CC globally each year, with more than half of them dying from the disease. Immunotherapy has rapidly become a cornerstone of cancer treatment, offering substantial improvements in survival rates and reducing treatment-related side effects compared to traditional therapies. For the past 25 years, chemoradiotherapy (CRT) has been the standard treatment for locally advanced CC (LACC). However, while adjuvant chemotherapy has failed to improve outcomes in LACC, the integration of neoadjuvant chemotherapy (NACT) with CRT, as well as chemoimmunoradiotherapy followed by consolidation immunotherapy, has transformed treatment strategies, demonstrating superior efficacy compared to CRT alone. In the first-line treatment of CC, adding pembrolizumab to platinum-based chemotherapy, either with or without bevacizumab, has significantly improved outcomes compared to platinum-based chemotherapy and bevacizumab alone. This review explores the current landscape of immunotherapy and biomarker advancements in CC. Furthermore, we discuss promising future directions, including the potential of personalized immunotherapy approaches and novel combination therapies to further enhance treatment efficacy and improve prognoses for patients with CC.

## Introduction

Cervical cancer (CC) is a disease that primarily affects young women. Although it is highly preventable, it remains the fourth most common cancer globally in both incidence and mortality [1]. In 2022, an estimated 661,021 new cases and 348,189 deaths were expected worldwide [1]. More than 70% of CC-related mortality occurs in countries with low and medium socioeconomic development, where CC ranks second in both incidence and mortality [1]. This disparity can be partially attributed to the unequal availability of prophylactic vaccination, inadequate screening programs, a resulting shift to more advanced disease stages at diagnosis, and a lack of appropriate treatment options throughout the course of therapy. In Europe, approximately 61,100 new cases of CC are diagnosed annually, with 25,800 related deaths each year [2]. The most critical risk factor for developing CC is infection with high-risk human papillomavirus (HR-HPV) types, mainly human papillomavirus (HPV)-16 and HPV-18 [3]. In developing countries, the disease is often diagnosed at stages III or IV [4–6], whereas in developed countries it is typically diagnosed at stages I or II, underscoring the urgent need for better-organized early detection programs [7, 8]. Evidence from a systematic review and meta-analysis conducted in high-income countries highlights the effectiveness of HPV vaccination programs [9]. After 5–8 years, a substantial decline in the prevalence of HPV-16 and HPV-18—the most common causes of CC—was observed. Specifically, the review demonstrated an 83% reduction in these high-risk HPV types among vaccinated girls aged 13–19 and a 66% decrease among vaccinated women aged 20–24.

CC therapy depends on the stage of the disease, and the main treatment options include surgery, radiotherapy, chemotherapy, and, more recently, immunotherapy and targeted therapy. Until the development of new therapeutic modalities, the success of treatment for recurrent and metastatic disease (r/m CC) was modest, with a median survival of less than 12 months in cases of distant metastasis [10]. The recent incorporation of immune checkpoint inhibitors (ICIs) into the therapeutic algorithm of CC has revolutionized cancer treatment and has become one of the most promising approaches in CC. Accordingly, identifying robust biomarkers to guide these novel treatments has become a priority, as discussed next.

## Biomarkers in CC

Biomarkers in oncology are biological indicators that reveal the presence, progression, or characteristics of cancer and are crucial for diagnosis, prognosis, treatment decisions, and monitoring therapeutic responses. They can be genetic, epigenetic, proteomic, glycomic, metabolomic, transcriptomic, or image-based. As with most other cancers, biomarkers in cervical lesions, including invasive CC, improve early detection, diagnosis, prognosis, and treatment response. HPV DNA testing is now considered the primary screening test for the detection of cervical lesions, rather than visual inspection with acetic acid (VIA) or cytology (Pap smear) (moderate-certainty level of evidence). This applies to all women over 30 years of age, irrespective of their risk for cervical lesions and subsequent CC [11].

The most common histologic subtype of CC is squamous cell carcinoma (SCC). Almost all (>95%) cervical SCCs are HPV-associated (positive). However, HPV-independent SCC, although rare, has been described in the literature [12]. Therefore, all cervical squamous lesions, including SCC, are classified into HPV-associated and HPV-independent subtypes [13]. The second most common histologic subtype of CC is adenocarcinoma. Similar to SCC, the new WHO classification of CC also recognizes HPV-associated and HPV-independent cervical adenocarcinomas [14]. In contrast to SCC, cervical adenocarcinomas appear to be less commonly associated with HPV infections [15, 16]. A subset of cervical adenocarcinomas may also be associated with an autosomal dominant syndrome called Peutz-Jeghers syndrome (#OMIM 175200). The syndrome is caused by germline mutations in the STK11 gene and is associated with various benign conditions (e.g., hamartomatous gastrointestinal polyps and mucocutaneous pigmentations) as well as an increased risk of various cancers [17].

HPV-associated and HPV-independent CC cannot be reliably distinguished based on morphologic criteria alone. Therefore, biomarkers are required for this subclassification. The most common biomarker is p16INK4a (p16), a surrogate marker for high-risk HPV infections (e.g., HPV-16, HPV-18). p16 expression in cervical lesions, including cancer, is tested by immunohistochemistry (IHC). Almost all HPV-associated CCs exhibit strong and diffuse nuclear and cytoplasmic p16 overexpression by IHC [18]. Strong and diffuse p16 positivity in cervical lesions indicates a transcriptionally active HPV infection; however, cases of p16- and HPV-negative cervical lesions, including CC, have been increasingly recognized and reported in the literature [19–21].

The clinical use of p16 IHC is frequently accompanied by Ki-67 IHC staining (MIB1 antibody), which indicates cellular proliferation. Combined (dual) p16/Ki-67 staining may be particularly useful in assessing the degree of cervical dysplasia, but Ki-67 expression alone is not sufficient, as it does not correlate with HPV infections. The combined use of p16/Ki-67 IHC is also useful in diagnosing and grading cervical glandular intraepithelial disease (precursors of cervical adenocarcinoma) [22]. In addition, HPV DNA testing and E6/E7 mRNA detection have become essential for identifying persistent high-risk HPV infections associated with CC development and progression (cervical intraepithelial lesions, CIN1–3) [23]. HPV DNA testing also exhibits superior sensitivity compared to cervical cytology alone.

Commonly observed genomic alterations in SCCs are those within the PI3K/MAPK and/or TGF-β signaling pathways [24]. The most frequently mutated genes in SCC are *ERBB3 (HER3), SHKBP1*, *CASP8, HLA-A,* and *TGFBR2* [24]. None of these genomic biomarkers has been approved as predictive for precision oncology purposes in CC patients, underscoring the need for new predictive biomarkers in the era of immunotherapy.

## Immunotherapy in CC

### ICIs in CC

**Figure 1.**
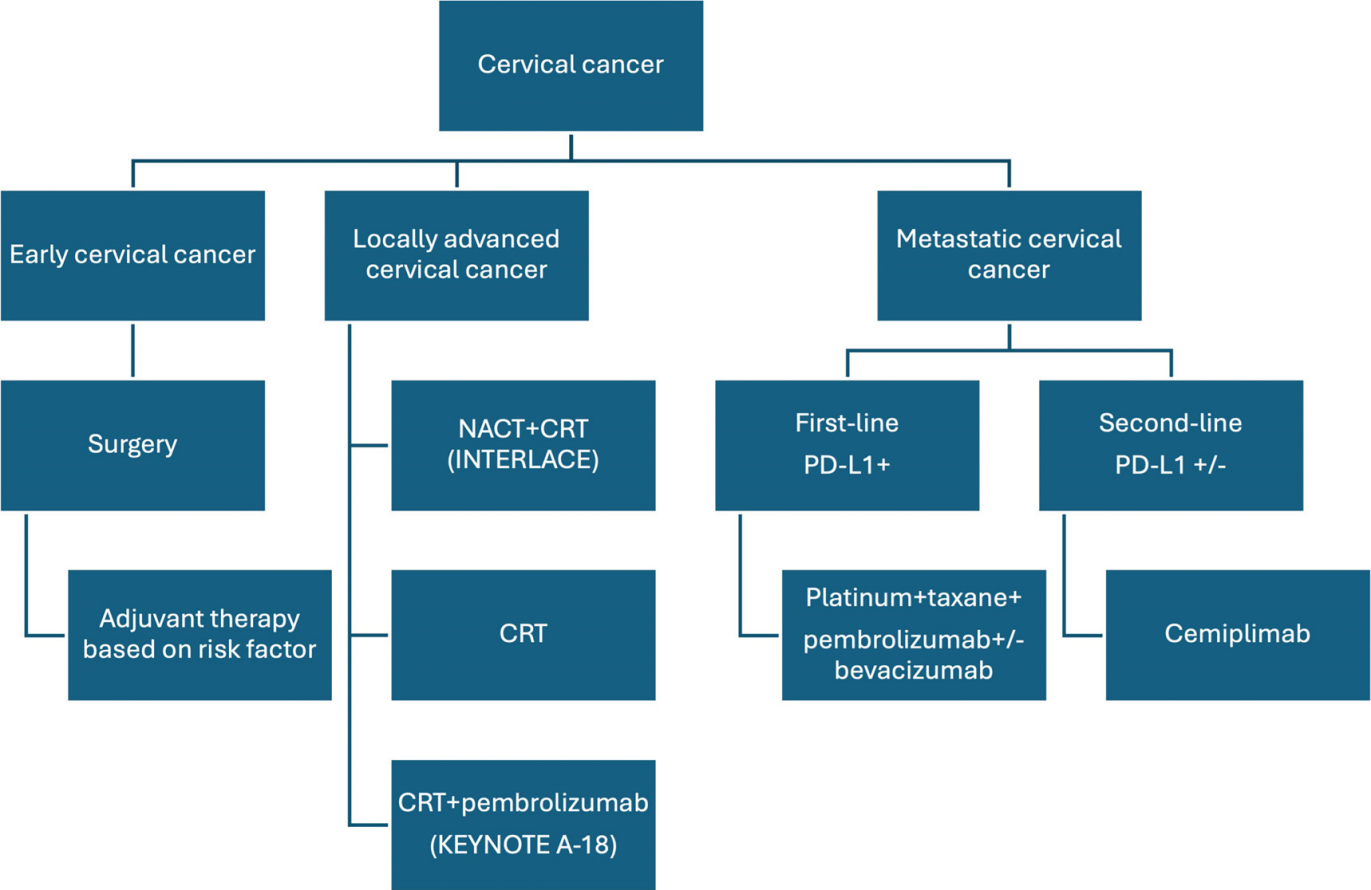
**The proposed treatment algorithms for invasive cervical cancer based on current evidence.** NACT: Neoadjuvant chemotherapy; CRT: Chemoradiotherapy; PD-L1: Programmed death receptor-1 ligand.

Immunotherapy with ICIs is an anti-cancer treatment directed against immune suppression in the tumor microenvironment, enabling the immune system to target and eliminate cancer cells. Specifically, T-cell activation requires two signals: antigen presentation via the major histocompatibility complex (MHC) on antigen-presenting cells (APCs) to the T-cell receptor, and co-stimulation by B7 on APCs binding to CD28 on T-cells to fully activate T-lymphocytes against a specific target. Conversely, when programmed death-ligand 1 (PD-L1), an inhibitory transmembrane protein, is present on the surface of APCs or cancer cells, it binds to programmed death receptor 1 (PD-1) on T-lymphocytes, suppressing T-cell activity and dampening the immune response. PD-L1 expression on cancer or immune cells has emerged as a predictive biomarker for ICIs in many, but not all, tumor types. In CC, PD-L1 expression assessed by IHC has been reported as positive in approximately 30%–70% of cases [25]. The introduction of ICIs has marked a significant advancement in the treatment of cancers, such as melanoma and lung cancer, revolutionizing the therapeutic landscape for these malignancies [26]. By targeting immune regulatory pathways like PD-1/PD-L1, ICIs have demonstrated notable efficacy across various cancer types, leading to their approval for multiple indications and transforming patient outcomes. The development of companion diagnostic tests (CDx) to identify individuals most likely to benefit from these therapies has further personalized and optimized their use in clinical practice. In CC, immunotherapy with ICIs has gained approval for second-line treatment, first-line treatment of recurrent or metastatic disease, and locally advanced CC (LACC; Figure 1).

### The role of immunotherapy in previously treated recurrent, metastatic CC

Before immunotherapy, the standard treatment for recurrent, persistent, or metastatic CC after first-line failure was chemotherapy [27]. There was little benefit from second-line or subsequent systemic therapies, with modest clinical efficacy: an overall response rate (ORR) of less than 20%, and median progression-free survival (PFS) and overall survival (OS) of 3.3 and 6.7 months, respectively [27, 28].

The first evidence for the clinical activity of ICIs in this setting was based on the phase Ib KEYNOTE-028 trial. This study examined the efficacy and safety of pembrolizumab (an anti-PD-1 monoclonal antibody) in patients with PD-L1-positive (≥1% by modified positive score) metastatic solid tumors, including 24 patients in the CC cohort (96% were SCC). The ORR was 17% (95% CI, 5%–37%), and the median duration of response (mDOR) was 5.4 months (95% CI, 4.1–7.5 months) [29]. KEYNOTE-158 revealed similar results [30]. The study investigated the safety and efficacy of pembrolizumab in patients with different metastatic tumors, regardless of PD-L1 status. Ninety-eight patients were included in the CC cohort; 82 samples were PD-L1 positive, defined as a combined positive score (CPS) ≥1%, with previously treated advanced CC. The ORR was 12.2% (95% CI, 8.0%–22.8%) in the whole cohort, and 14.6% in the patient subgroup with PD-L1-positive tumors. The median PFS was 2.1 months (95% CI, 2.1–2.2 months), and OS was 9.3 months (95% CI, 7.6–11.7 months). The safety profile was consistent with that observed for pembrolizumab in other tumor types [30]. Based on these results, the FDA approved pembrolizumab for patients with persistent, r/m CC whose tumors express PD-L1 ≥1%. ICI and antibody-drug conjugate (ADC) regimens approved to date by the FDA and EMA for the treatment of CC are summarized in Table 1.

**Table 1:** Summary of clinical trials with FDA-approved immunotherapy and ADC regimens in the treatment of cervical cancer

**Drug**	**Trial**	**Patient population**	**Treatment	**ORR	**mPFS**	**mOS	**Approval year**
Bevacizumab	Phase III GOG 240 [27]	r/m or persistent CC, no prior CHT	CHT + bevaci zumab vs CHT	45% vs 34%	8.2 vs 6.0 months (HR 0.68)	16.8 vs 13.3 months (HR 0.77) 24.5 vs 16.8 months (HR 0.64) in pts not treated with prior CRT	2014
Pembrolizumab	Phase II KEYNOTE-158 [30]	r/m CC (PD-L1 [CPS] ≥ 1) with disease progression on prior systemic treatments	Pembrolizumab for 35 cycles or disease progression or unacceptable toxicity	14.6% (95% CI, 7.8%–24.2%)	4.1 month (95% CI, 2.4–4.9 months)	23.5 month (95% CI, 13.5 months to NR)	2018
Vedotin	Phase II Innova TV204 [79] Phase III Innova TV 301 [80]	r/m CC with disease progression on prior systemic treatment	Tisotumab Vedontin Tisotumab Vedontin vs CHT	24% 17.8% vs 5.2%	Not reported 4.2 vs 2.9 months (HR 0.67)	Not reported 11.5 vs 9.5 months (HR 0.70)	FDA accelerated approval 2021 2024
Pembrolizumab	Phase III KEYNOTE-826 [41]	r/m or persistent CC, no prior CHT, known PD-L1 status prior to randomization	CHT +/− bevacizumab (investigator choice) + pembrolizumab or CHT +/− bev acizumab (investigator choice) + placebo	65.9% vs 50.8%	10.4 vs 8.2 months (HR 0.65)	24.4 vs 16.5 months (HR 0.64)	2021
Pembrolizumab	Phase III KEYNOTE-A-18 [87]	Newly diagnosed, high-risk, stage IB2–IIB with node-positive disease or stage III and IVA irrespective of the nodal state (FIGO 2014)	Pembrolizumab + CRT vs Placebo + CRT	Not reported	NR in either group (rates at 24-month 68% vs 57%) (HR 0.70)	36-month overall survival 82.6% vs 74.8% (HR 0.67)	2024
Cemiplimab	Phase III EMPOWER-CERVICAL 1/GOG-30 16/ ENGOT-cs9 [105]	r/m CC with disease progressionon prior systemic treatment	Cemiplimab vs investigator’s choice chemotherapy	16.4% vs 6.3%	(HR 0.75; 95% CI, 0.63–0.89; *P* < 0.001)	12.0 vs 8.5 months (HR 0.69)	FDA BLA voluntarily withdrawn January 2022 EMA approved 2022

R/m or persistent CC: Recurrent, metastatic or persistent cervical cancer; CHT: Chemotherapy; CRT: Chemoradiation; Pts: Patients; ADC: Antibody-drug conjugate; ORR: Overall response rate; PFS: Progression-free survival; OS: Overall survival; PD-L1: Programmed cell death ligand-1; EMA: European Medicines Agency; FDA: Food and Drug Administration; BLA: Biologics License Application; NR: Not reached; CPS: Combined positive score; FIGO: International Federation of Gynecology and Obstetrics; mOS: Median overall survival; mPFS: Progression-free survival; HR: Hazard ratio.

Beyond pembrolizumab, another ICI, cemiplimab, has demonstrated improved survival in second-line treatment. Cemiplimab, a programmed cell death-1 receptor monoclonal antibody, significantly improved PFS and OS compared to chemotherapy in the phase III randomized study EMPOWER-Cervical 1/GOG-3016/ENGOT-cx9 [27]. This study enrolled patients with r/m CC—either SCC or adenocarcinoma—who had progressed after first-line platinum-based chemotherapy, regardless of PD-L1 status. In the overall trial population, median OS was longer in the cemiplimab group (12.0 months vs 8.5 months; hazard ratio [HR] 0.69; 95% CI, 0.56–0.84; *P* < 0.001), with consistent benefits in both histologic subgroups. PFS was also longer in the cemiplimab group (HR 0.75; 95% CI, 0.63–0.89; *P* < 0.001). Grade ≥3 adverse events were less frequent in the cemiplimab group compared to the chemotherapy group. Cemiplimab has been approved by the EMA for r/m CC, regardless of PD-L1 status, in patients who have progressed on platinum-based chemotherapy.

These trials established single-agent anti-PD-1 therapy as a new standard after chemotherapy failure, setting the stage for exploring combination immunotherapies.

#### Is dual immunotherapy better?

Several phase I/II trials have investigated anti-PD-(L)1 in combination with either anti-CTLA-4—blocking cytotoxic T-lymphocyte antigen-4 (CTLA-4)—or anti- T-cell immunoreceptor with Ig and ITIM (TIGIT)—blocking TIGIT domains—in advanced CC. TIGIT is an inhibitory target molecule expressed on T cells and natural killer cells [31].

CheckMate 358 is an open-label, multicohort phase I/II trial that evaluated the efficacy of nivolumab, alone or in combination with ipilimumab, in patients with virus-related tumors [32]. Patients with HPV-negative tumors were excluded from the study. The CC cohort enrolled patients with r/m SCC of the cervix and up to two prior systemic therapies. Patients were randomized into three groups: nivolumab monotherapy 240 mg, nivolumab plus ipilimumab every six weeks (Nivo 3 mg/kg + Ipi 1 mg/kg), and nivolumab plus ipilimumab every three weeks for four cycles, followed by nivolumab every two weeks (Nivo 1 mg/kg + Ipi 3 mg/kg). According to preliminary findings, nivolumab showed durable anti-tumor responses, and the combination of nivolumab and ipilimumab also demonstrated promising clinical activity. Nivolumab monotherapy yielded an ORR of 26%, median PFS (mPFS) of 5.1 months, and median OS (mOS) of 21.6 months. The two combination arms showed ORRs of 31% (Nivo3 + Ipi1) and 40% (Nivo1 + Ipi3), with the Nivo1 + Ipi3 arm achieving the longest mPFS of 7.2 months and mOS of 24.7 months, albeit with more pronounced toxicity.

Cadonilimab is a novel bispecific ICI that targets PD-1 and CTLA-4. By targeting both pathways, cadonilimab may provide synergistic effects while minimizing side effects compared with the combination of two separate monoclonal antibodies (mAbs). Cadonilimab was approved in China in 2022 for the second-line treatment of r/m CC. The approval was based on the positive results of a phase II clinical study, which assessed the efficacy and safety of cadonilimab in patients with r/m CC who had progressed on or after two or fewer prior chemotherapy regimens, with or without bevacizumab, regardless of PD-L1 status [33]. The primary endpoint was ORR, which was 33%; the median PFS and OS were 3.75 months and 17.51 months, respectively. Subgroup analysis showed that in PD-L1-positive patients, the ORR was 43.8%, with a median PFS of 6.34 months. The median OS was not reached (NR). The incidence rate of grade ≥3 adverse events was 27%, suggesting that cadonilimab may have a more favorable safety profile than dual therapies combining separate PD-1 and CTLA-4 inhibitors. A phase III trial is ongoing and is exploring the combination of cadonilimab with concurrent chemoradiotherapy (CCRT) for the treatment of LACC [34].

Balstilimab (PD-1 inhibitor) and zalifrelimab (CTLA-4 inhibitor) have been investigated in patients with r/m CC who had progressed on first-line therapy [35]. The ORR was 25.6% in the overall cohort and 32.8% in the patient subgroup whose tumors expressed PD-L1 ≥1%. In patients with squamous cell histology, the ORR was 32.6%. Further investigation of the balstilimab and zalifrelimab combination in this setting is ongoing.

Tiragolumab, an anti-TIGIT antibody, in combination with atezolizumab was investigated in the SKYSCRAPER-04 phase II trial (NCT04300647). The results of the study did not show an improvement in ORR compared to patients treated with atezolizumab alone [36]. The combination of pembrolizumab and vibostolimab (an anti-TIGIT antibody) was evaluated in the phase I KEYVIBE-001 trial (NCT02964013) and the phase II KEYVIBE-005 trial (NCT50007106), but it did not improve ORR, PFS, or OS compared to pembrolizumab alone in metastatic CC with PD-L1 CPS ≥1 [37, 38]. Given these results, the combination has no future in treating patients with PD-L1–positive recurrent CC. Tislelizumab (anti-PD-1) and ociperlimab (anti-TIGIT) are currently being investigated in combination for r/m CC as part of the phase II AdvanTIG-202 trial. The study has begun enrollment, and recruitment is ongoing [39].

Bintrafusp alfa is an innovative immunotherapy agent representing a bifunctional fusion protein. It combines two mechanisms: it acts as a “trap” for TGF-β, a key cytokine that promotes tumor growth, and simultaneously blocks the PD-L1 protein. In a phase II trial involving 146 patients with r/m CC who had experienced disease progression during or after platinum-based chemotherapy, the confirmed ORR was 21.9%, with manageable toxicity [40].

The lack of superiority of any investigated immunotherapy combinations over the mono-immunotherapy approach and their combinations with existing treatment modalities highlights the complexity of the tumor microenvironment. This suggests that further research is needed to determine the optimal timing and which combined approaches may offer benefits. It also underscores the importance of further evaluation of biomarkers to identify subgroups that may benefit from combined approaches to immunotherapy. Additionally, further studies are needed to determine the safety and efficacy of these combinations in patients who have already been exposed to immunotherapy, as previous research has been conducted in immunotherapy-naive patients.

#### Immunotherapy as a first-line treatment for recurrent, metastatic CC

In 2021, the FDA approved pembrolizumab in conjunction with chemotherapy, with or without bevacizumab, for patients with r/m CC with PD-L1 expression (CPS ≥1), based on the results of the KEYNOTE-826 study (Table 1). The KEYNOTE-826 trial was designed to assess the effectiveness of pembrolizumab as a first-line treatment for patients with r/m CC, regardless of PD-L1 expression, when combined with chemotherapy, with or without bevacizumab. In advanced CC, PD-L1 positivity (CPS ≥1) was reported in 83.7% of patients in the KEYNOTE-158 trial and 88.8% in the KEYNOTE-826 trial [30, 41]. The median PFS for patients with PD-L1 ≥1% (CPS ≥1) was 8.2 months in the placebo group and 10.4 months in the pembrolizumab group (HR 0.62; 95% CI, 0.50–0.77; *P* < 0.001). In the intent-to-treat (ITT) group, PFS was 10.4 and 8.2 months, respectively (HR 0.65; 95% CI, 0.53–0.79; *P* < 0.001). Pembrolizumab demonstrated a statistically significant and clinically meaningful survival benefit. At 24 months of follow-up, the OS rate in the PD-L1 >1 group was 53% with pembrolizumab compared to 41.7% with placebo (HR 0.64; 95% CI, 0.50–0.81; *P* < 0.001). In the ITT population, the OS rates were 50.4% and 40.4% for pembrolizumab and placebo, respectively (HR 0.67; 95% CI, 0.54–0.84; *P* < 0.001). The safety profile showed slightly higher rates of anemia and neutropenia in the pembrolizumab group [41]. After a median follow-up of over three years, pembrolizumab continued to extend OS and PFS in the CPS ≥1 and ITT populations across all subgroups (determined by histologic type, prior use of bevacizumab, and chemoradiotherapy (CRT) treatment). The median OS in the PD-L1 CPS ≥1 population was similar for both the bevacizumab-treated (HR 0.62; 95% CI, 0.45–0.87) and bevacizumab-untreated (HR 0.67; 95% CI, 0.47–0.96) subgroups. Pembrolizumab resulted in a median OS of 24.4 months for squamous histology compared to 14.2 months with placebo (HR 0.60; 95% CI, 0.46–0.79). For non-squamous histology, median OS was NR in the pembrolizumab group compared to 23.5 months in the placebo group (HR 0.70; 95% CI, 0.41–1.20). The HR for OS was 0.56 (95% CI, 0.39–0.81) in patients with previous CRT and 0.72 (95% CI, 0.52–1.00) in those without prior CRT. Additionally, the HR for OS favored the pembrolizumab group across all ITT population subgroups. More than three years of follow-up revealed no additional safety concerns [42].

In line with the benefits observed with bevacizumab in KEYNOTE-826, the BEATcc trial, a phase III study, evaluated the addition of atezolizumab, an anti-PD-L1 ICI, to a standard regimen of chemotherapy and bevacizumab in patients with r/m CC, irrespective of PD-L1 status. Median PFS for the atezolizumab and standard therapy groups was 13.7 and 10.4 months, respectively (HR 0.62; 95% CI, 0.49–0.78; *P* < 0.0001), while median OS was 32.1 and 22.8 months (HR 0.68; 95% CI, 0.52–0.88; *P* ═ 0.0046). In the atezolizumab group, 79% of patients experienced adverse events of grade ≥3, compared to 75% in the control group [43].

Cadonilimab has shown promising results in the first-line treatment of r/m CC in trials conducted in China. The effectiveness of cadonilimab in combination with standard chemotherapy, with or without bevacizumab, as a first-line treatment for r/m CC was evaluated in the phase II trial COMPASSION-13 [44]. With an ORR of 92.3% in patients who received cadonilimab, chemotherapy, and bevacizumab, this trial showed encouraging findings. Another study reported an ORR of 71.4% with cadonilimab in PD-L1-negative patients, with an even higher response rate of 80% [45]. In real-world settings, cadonilimab demonstrated an ORR of 43% and a disease control rate (DCR) of 77.4% [46]. The phase III COMPASSION-16 trial further evaluated cadonilimab in combination with first-line chemotherapy, with or without bevacizumab, in patients with persistent, recurrent, or metastatic CC [47]. The addition of cadonilimab significantly improved PFS and OS compared to the placebo group. Median PFS was 12.7 months in the cadonilimab group vs 8.1 months in the placebo group (HR 0.62; 95% CI, 0.49–0.80; *P* < 0.0001). Median OS was NR in the cadonilimab group (27.0 months to not estimable) compared to 22.8 months in the placebo group (HR 0.64; 95% CI, 0.48–0.86; *P* ═ 0.0011). Together, these data suggest that adding PD-1/CTLA-4 bispecific immunotherapy to first-line chemotherapy (± bevacizumab) can substantially improve outcomes, offering a potential new treatment option pending broader regulatory review.

### The role of immunotherapy for LACC

Over the last twenty years, the mainstay of care for LACC has been CCRT followed by brachytherapy. CCRT demonstrated a substantial five-year survival advantage of about 6% (HR 0.81, *P* < 001) when compared to radiotherapy alone, according to five major randomized trials that investigated the addition of chemotherapy to pelvic radiation. CRT also improved disease-free survival and reduced both local and distant recurrence [48–52]. Despite these advances, recurrence rates remain high—about 40% of patients experience a recurrence of the disease within five years—and five-year OS remains around 65%–70% [53]. Significant progress has been made in the treatment of LACC through techniques, such as image-guided radiation therapy (IGRT) and intensity-modulated radiation therapy (IMRT)/volumetric arc therapy (VMAT). These advances have potentially improved survival and reduced treatment-related morbidity, though without a significant impact on outcomes at the global level [54].

New strategy trials are ongoing in locoregional disease, including studies using PD-L1 ICIs (atezolizumab and durvalumab) or PD-1 ICIs (nivolumab, pembrolizumab) in the context of LACC. Pembrolizumab in conjunction with CRT is being investigated in the ENGOT-cx11/GOG-3047/KEYNOTE-A18 study, a phase III clinical trial, for the treatment of high-risk LACC—stage IB2–IIB with node-positive disease or stage III–IVA irrespective of nodal status—with histologically proven carcinoma (FIGO 2014). The results showed that as compared to CRT alone, pembrolizumab significantly increased PFS and OS. The median follow-up period was 17.9 months, and the PFS was 67.8% in the pembrolizumab–CRT group and 57.3% in the placebo–CRT group (HR 0.70; 95% CI, 0.55–0.89; *P* ═ 0.0020). Neither group reached the mOS; the 36-month OS was 74.8% in the placebo–CRT group and 82.6% in the pembrolizumab–CRT group (HR 0.67; 95% CI 0.50–0.90; *P* ═ 0.0040). Hematological toxicity was the most common adverse event, with no noticeable difference between the two groups [49]. According to the findings of the ENGOT-cx11/GOG-3047/KEYNOTE-A18 study—the first positive randomized phase III trial in this patient population since 1999—pembrolizumab in combination with CRT could become the new standard of care for patients with LACC. In 2024, the FDA approved pembrolizumab combined with CRT for patients with LACC (stages III and IV, FIGO 2014) (Table 1). The CALLA trial is a phase III, multicenter trial evaluating the efficacy of combining durvalumab with CRT for patients with LACC (adenocarcinoma, squamous, or adenosquamous), including stage IB2–IIB with lymph node-positive disease or stage ≥III irrespective of nodal status. However, the trial results did not demonstrate a substantial improvement in PFS, which was the study’s primary endpoint. Durvalumab showed a 12-month PFS of 76%, compared to 73.3% for placebo (HR 0.84; 95% CI, 0.65–1.08; *P* ═ 0.17). There was no significant difference in toxicity between the two groups [55]. Possible reasons for the negative results of the CALLA study include the short follow-up period, the use of PFS as the primary endpoint, inclusion of a broader population with locally advanced disease, and the fact that patients in KEYNOTE-A18 represented a higher-risk group. Additionally, the study did not identify or focus on subgroups that might derive greater benefit (e.g., those with high levels of PD-L1 expression). The results of the CALLA study highlight the need for better-defined biomarkers and more precise patient selection to identify those who may benefit from immunotherapy in combination with standard treatment options. Nevertheless, we eagerly await the most important outcome of this trial: the OS results.

The NiCOL phase 1 trial aims to assess the safety and recommended trial dose of concurrent nivolumab with definitive CRT, followed by nivolumab as maintenance treatment, in 16 patients with LACC. Initial results were positive: the ORR was 93.8% (95% CI: 69.8%–99.8%), and the two-year PFS was 75% (95% CI: 64.2%–100%) [56]. The promising results and safety profile of nivolumab in this study highlight the need for further research to investigate its role in the treatment of LACC.

The ATEZOLACC trial is a phase II, open-label study examining the effectiveness of atezolizumab administered concurrently with and after CCRT in high-risk patients (IB1–IIA with positive nodes, stages IIB–IVA, and any stage with para-aortic positive lymph nodes). The primary endpoint is PFS, and the first results are awaited [57].

Another approach being tested is consolidation immunotherapy after completing chemoradiation. The ATOMICC trial is a double-blind, phase II study with a slightly different objective: investigating the efficacy of dostarlimab as a consolidation treatment for patients with locally advanced, high-risk CC who responded partially or completely to chemoradiation. The goal is to evaluate whether dostarlimab can reduce recurrence and improve survival compared to follow-up without additional treatment after response to CRT. Recruitment is closed, and results are awaited [58].

#### Is it time for neoadjuvant immunotherapy in CC?

According to the INTERLACE trial, patients with LACC who received induction chemotherapy followed by standard chemoradiation had impressive outcomes compared to those who received standard chemoradiation alone. Following a median follow-up of 67 months, the five-year PFS was 72% in the induction chemotherapy plus CRT group and 64% in the CRT-alone group (HR 0.65; 95% CI, 0.46–0.91; *P* ═ 0.013). The five-year OS was 80% in the induction chemotherapy group vs 72% in the CRT-alone group (HR 0.60; 95% CI, 0.40–0.91; *P* ═ 0.015) [59].

Neoadjuvant immunotherapy is a developing strategy in the treatment of many cancers, and this approach could also lead to similar breakthroughs in CC [60].

Neoadjuvant camrelizumab plus chemotherapy is being investigated in patients with LACC (IB3 to IIB/IIIC1, PD-L1 positive, with a tumor diameter ≥4 cm) in the NACI study (NCT04516616), a single-arm, phase II clinical trial. Patients who had a complete or partial response underwent major surgery, whereas those with progressive or stable disease received concomitant CRT. At the data cutoff, the median follow-up was 11 months, and the ORR was 98% (95% CI, 92–100). No serious adverse events were reported [61]. Immune-based neoadjuvant chemotherapy (NACT) is currently being studied in two additional trials. The first is the NCT04799639 trial, which recruited patients with stage IB3 and IIA2 CC to receive NACT consisting of paclitaxel, cisplatin, and sintilimab (a PD-1 inhibitor) for three cycles prior to undergoing radical surgery. The ORR was 95%, and as of the data cutoff in February 2024, 33% of patients had achieved a pathological complete response. The anticipated date of study completion is March 2026 [62]. Notably, all these neoadjuvant immunotherapy trials are reporting high response rates (≈95% or higher), suggesting the potential of this strategy to shrink tumors prior to surgery. However, longer follow-up is needed to determine whether this translates into improved survival.

The MITO CERV 3 trial (NCT04238988), a phase II, single-arm, multicenter study, is the second to investigate the use of pembrolizumab in combination with carboplatin and paclitaxel as neoadjuvant therapy for LACC (stages IB2–IIB, PD-L1-positive tumors). Maintenance therapy with pembrolizumab was administered to patients after surgery if there was no disease progression. The study is ongoing, and results are awaited [63].

Neoadjuvant immunotherapy in combination with chemotherapy shows promising antitumor activity with manageable safety profiles, and this approach may improve rates of complete pathological responses and event-free survival in patients with LACC. Future research should focus on identifying which patients are most likely to benefit based on predictive biomarkers, as well as establishing the optimal timing, sequencing, and integration of these therapies with current neoadjuvant or concomitant treatment regimens.

### New treatment approaches

Although treatment with ICIs has been most extensively studied in CC with very promising results, numerous alternative approaches and therapeutic strategies are under investigation. The two most promising approaches are cell-based therapies and therapeutic vaccinations [64].

#### Therapeutic vaccines

Therapeutic vaccines for HPV represent an innovative approach to treating HPV infections and related diseases, such as CC and other malignancies. Therapeutic vaccinations aim to cure HPV-caused lesions or infections that have already occurred, whereas prophylactic vaccines are intended to prevent HPV infection. Therapeutic vaccines most commonly target E6 and E7 oncoproteins, which are involved in the harmful process of cellular transformation and stimulate T-cell immunity (especially CD8+ cytotoxic T cells) to kill infected or malignant cells. More than 20 therapeutic HPV vaccine candidates are currently in various phases of research, with several undergoing clinical trials. Some therapeutic vaccines, such as VGX-3100, are in the later phases of clinical research and show efficacy in treating precancerous CIN 2/3 lesions, but none have yet received widespread regulatory approval for the treatment of cervical lesions or cancer [65]. The effectiveness and safety of PDS0101, an HPV-specific T-cell-activating immunotherapy, are being assessed in the phase II IMMUNOCERV trial (NCT04580771), in combination with CRT for patients with locally advanced HPV-associated CC (stage IB3 to IVA). Initial findings indicated a favorable safety profile, an 88% complete metabolic response rate on post-treatment imaging, an 89% DFS after one year, and 100% OS [66]. Further updates on long-term outcomes are awaited.

Therapeutic vaccines hold great promise for patients with advanced or recurrent CC, as well as for those with precancerous lesions caused by persistent HPV infection. They have the potential to become a key part of the treatment for HPV-related diseases, significantly reducing the incidence and mortality of HPV-related malignancies.

#### ICI + vaccine combinations

The combination of ICIs and therapeutic vaccines represents an innovative and promising approach to the treatment of CC, and the effectiveness of this combination is currently being studied in clinical trials. Atezolizumab plus the HPV16 vaccine VB10.16 showed promising results in HPV16-positive metastatic CC in a phase II study, with a preliminary median OS greater than 25 months [67]. Pembrolizumab combined with another vaccine, GX-188E, has also shown manageable toxicity and encouraging activity in HPV16- and 18-positive metastatic CC [68]. Likewise, cemiplimab with the HPV16 vaccine ISA101b demonstrated clinical benefit in HPV16-positive metastatic CC in a phase II trial [69].

#### Chimeric antigen receptor (CAR) T-cell therapy

Mesothelin, a membrane protein highly expressed in CC, represents a promising target for immunotherapy. Preclinical studies have shown that anti-mesothelin CAR-NK cells exhibit significant cytotoxicity against CC cells [70]. Additionally, HPV-specific targeting shows promise, particularly against the viral oncoproteins driving malignancy. A preclinical study reported that E6-targeted CAR-T cells effectively kill HPV16-positive CC cells, highlighting a strategy to exploit viral antigen specificity [71]. Clinical trials for CAR T-cell therapy in CC are still in the early stages. More clinical data are needed to establish its safety and effectiveness in this context [72].

#### ADCs

Apart from immunotherapy, a new and promising treatment class in oncology is ADCs. These biopharmaceuticals combine mAbs with chemotherapeutic agents, enabling the direct delivery of cytotoxic drugs to cancer cells while sparing healthy tissues, thus reducing systemic toxicity [73]. Targeting highly expressed antigens in CC offers a promising therapeutic strategy, particularly through the use of ADCs. Potential targets under investigation include folate receptor alpha (FRα), expressed in approximately 40% of cases; tissue factor (TF), found in about 77%; human epidermal growth factor receptor 2 (HER2), present in 1%–12%; trophoblast cell surface antigen 2 (TROP2), highly expressed in around 85%; and epidermal growth factor receptor (EGFR), observed in 70%–90% of cases [74–78].

Several trials are examining the safety and effectiveness of ADCs in CC. Tisotumab vedotin, an antibody-drug conjugate that targets TF, was the focus of the innovaTV 204 trial, a single-arm, phase II clinical trial assessing its effectiveness in patients with r/m CC who had undergone up to two previous systemic therapies. The ORR was approximately 24% and was observed across histological subtypes (squamous and nonsquamous). Grade 3/4 adverse events were reported in 28% of cases, with alopecia, nausea, conjunctivitis, and fatigue being the most prevalent adverse effects [79]. The findings of this study demonstrated that tisotumab vedotin has clinically meaningful activity in heavily pretreated CC patients. In September 2021, the FDA approved it for use in treating r/m CC in patients whose disease progresses during or after chemotherapy (Table 1).

The innovaTV 301/ENGOT-cx12/GOG-3057, a phase III clinical trial, evaluated the efficacy of tisotumab vedotin in treating r/m CC following progression on first-line systemic therapy. Results demonstrated that tisotumab vedotin significantly improved PFS and OS compared to chemotherapy, with a median OS of 11.5 months vs 9.5 months for chemotherapy (HR 0.70; 95% CI, 0.54–0.89; two-sided *P* ═ 0.004). The median PFS was 4.2 months with tisotumab vedotin vs 2.9 months with chemotherapy (HR 0.67; 95% CI, 0.54–0.82; two-sided *P* < 0.001). Grade ≥3 treatment-related side effects were less common with tisotumab vedotin (29.2%) than with chemotherapy (45.2%) and were generally tolerable [80]. The FDA approved tisotumab vedotin in April 2024 for the treatment of patients with r/m CC whose disease progresses during or following chemotherapy (Table 1).

In the DESTINY-PanTumor02 study, a multicenter, phase II, open-label clinical trial, patients with advanced or metastatic solid tumors that had progressed on prior therapies were evaluated for the safety and effectiveness of trastuzumab deruxtecan (T-DXd), an ADC that targets HER2. The ORR for all patients was 37.1%; for CC patients, it was 50%. The mDOR was 9.8 months, and PFS was seven months. In IHC 3+ cases, the ORR was 75% and PFS was NR. Drug-related side effects of grade ≥3 were observed in 40.8% of patients [81]. Based on results from the DESTINY-PanTumor02 trial and supporting data from studies in lung and colorectal cancer, the FDA, in August 2024, granted tumor-agnostic approval for fam-T-DXd-nxki [82, 83]. The indication includes patients with HER2-positive (IHC 3+) unresectable or metastatic solid tumors who have received prior systemic therapy.

Sacituzumab govitecan (SG) is an ADC that combines an anti-Trop-2 monoclonal antibody with the cytotoxic agent SN-38, a topoisomerase I inhibitor, allowing targeted delivery of chemotherapy to Trop-2–expressing tumors [84]. CC often exhibits high Trop-2 expression, making SG a plausible candidate for treatment [85]. Patients with advanced CC who are resistant or intolerant to chemotherapy are being recruited for the EVER-132-003 trial, a multicenter, multi-cohort phase II clinical study that includes SG in the treatment of various solid malignancies. An interim review revealed a 50% ORR and no new safety concerns [72]. For r/m CC patients, SG may represent a new targeted treatment option for those who overexpress Trop-2.

As with other cancers, the role of ADCs in CC treatment is promising. ADCs could revolutionize the treatment landscape for CC by offering a more precise and effective option and may provide new hope as first- or second-line treatments, especially for patients with limited responses to conventional approaches. Combining ADCs with existing therapies—such as immunotherapy, radiation, or other targeted treatments—could enhance their effectiveness, and future research into these combinations may provide valuable insights. As novel therapies like ADCs emerge, identifying biomarkers that predict response to various treatments becomes increasingly critical.

### Association between immune biomarkers and outcomes in CC: Implications for immunotherapy

**Figure 2.**
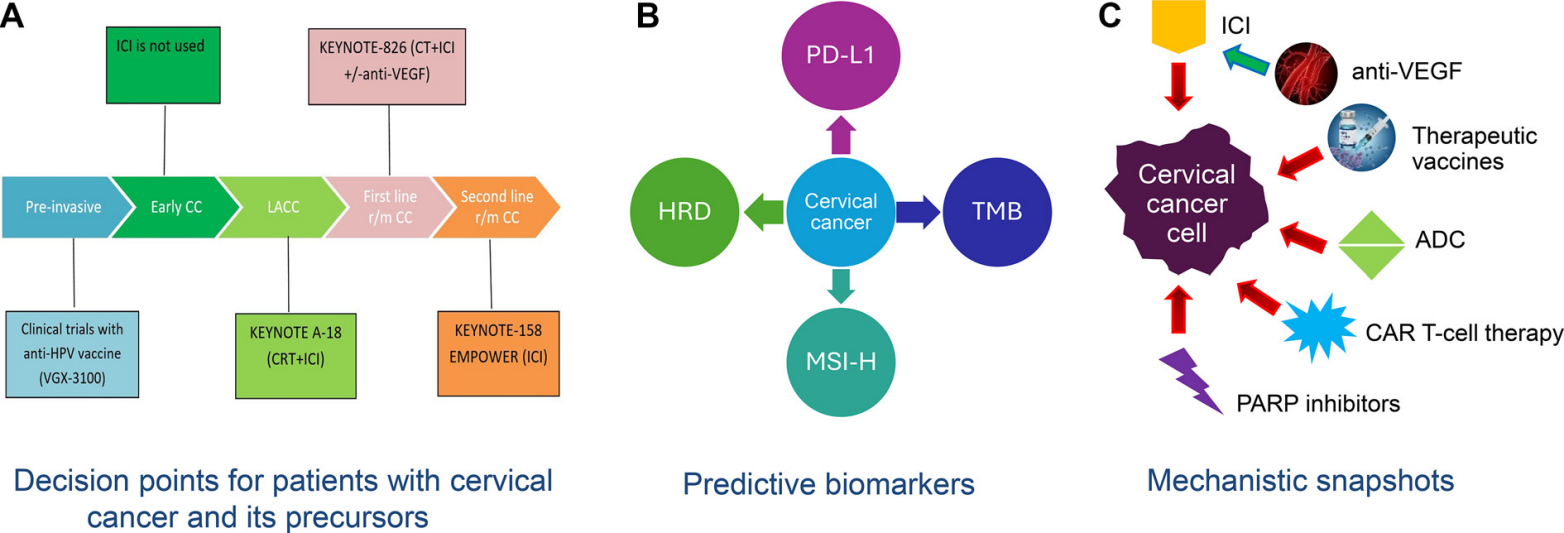
**Potential biomarkers for immunotherapy in cervical cancer. Red arrows indicate inhibitory, while the green arrow (anti-VEGF therapies) indicates an enhancing effect (image C).** TMB: Tumor mutational burden; MSI-H: Microsatellite instability high; PD-L1: Programmed death receptor-1 ligand; HRD: Homologous recombination deficiency; ICI: Immune checkpoint inhibitors; PARP: Poly (ADP-ribose) polymerase.

Biomarkers play a critical role in predicting clinical outcomes in CC, especially in the context of immunotherapy (Figure 2). Several studies have highlighted predictive and prognostic biomarkers that can help guide treatment decisions.

PD-L1 expression, assessed by IHC, is the most widely used biomarker to predict response to immunotherapy [86]. While the general literature reports PD-L1 expression in approximately 30%–70% of CCs, clinical trials have demonstrated even higher positivity rates, particularly in advanced-stage disease [25]. It is also considered a potential surrogate marker for HPV infection. In the KEYNOTE-826 study, which evaluated pembrolizumab in recurrent or metastatic CC, PD-L1 expression was observed in 89% of tumors [41]. Similarly, the KEYNOTE-A18 trial, which investigated treatment in LACC, reported PD-L1 expression in 95% of patients [87]. PD-L1 expression is also a predictive biomarker of response to pembrolizumab in both first-line and later-line treatment of CC. In first-line treatment, subgroup analyses demonstrated improved PFS and OS in patients with PD-L1–positive tumors compared to those without expression [41]. However, in LACC, adding pembrolizumab to CRT provided clinical benefit regardless of PD-L1 expression, suggesting that PD-L1 may not be an optimal biomarker for ICI response in CC [87].

The number of mutations found in the coding regions of the cancer genome is known as tumor mutational burden (TMB). It can serve as a predictive biomarker for how likely a cancer is to evoke an immune response, influencing its potential sensitivity to immunotherapy with ICIs. Higher TMB often correlates with a more robust response to certain ICIs [88, 89]. The prognostic value of TMB has been demonstrated in CC [90], with patients showing significantly improved five-year survival compared to those with low TMB. In the KEYNOTE-158 study, which evaluated immunotherapy in advanced solid tumors, including CC, TMB was investigated as a potential biomarker of immunotherapy response [91]. The study found that high TMB (≥10 mutations per megabase) was present in 13% of all solid tumor patients analyzed. Small cell lung cancer (33%) and CC (16%) showed the highest prevalence of high TMB. Clinical implications of TMB have also been observed in another study on squamous CC [92], where the five-year survival rate was considerably higher for patients with high TMB than for those with low TMB. Interestingly, the threshold for high vs low TMB was based on the median TMB in the cohort [92]. Among the clinical data, 69% of patients had T1 or T2 CC, while metastatic status was unknown for 59% of patients. In a retrospective study of 44 patients with FIGO IB–IVA squamous LACC treated with CRT or radiotherapy, high TMB combined with low CD8+ tumor-infiltrating lymphocyte (TIL) density was associated with poorer OS, PFS, and distant metastasis-free survival (*P* ═ 0.012, *P* ═ 0.27, and *P* ═ 0.047, respectively) [93]. Treatment intensification may be beneficial for these patients, including chemoimmunoradiotherapy and consolidation immunotherapy, as outlined in the KEYNOTE-A18 protocol.

Microsatellite instability (MSI) implies genetic hypermutability due to defects in the DNA mismatch repair (MMR) system. Tumors that exhibit high levels of MSI, referred to as MSI-High (MSI-H), tend to accumulate numerous mutations throughout repetitive DNA sequences known as microsatellites [94–96]. MSI-H cancers typically exhibit strong responses to immunotherapies, such as ICIs, due to their increased neoantigen load. The highest prevalence of MSI-H has been demonstrated in colorectal, endometrial, and gastric adenocarcinomas. A retrospective study investigated the frequency of MMR deficiency/high MSI (MMRd/MSI-H) and its role as a predictive biomarker of response to immunotherapy in gynecological cancers, including CC [97]. MMRd/MSI-H was identified in approximately 10% of gynecologic cancers with SCC morphology. Among patients treated with ICIs, a response was observed in eight of 37 cases (22%), suggesting that MMRd/MSI-H may serve as a potential biomarker for predicting immunotherapy response in CC.

Homologous recombination deficiency (HRD) refers to the inability of cells to properly repair double-strand DNA breaks through the homologous recombination pathway. HRD causes genetic instability and the accumulation of mutations [98, 99]. Cancers exhibiting HRD often rely on alternative, less accurate DNA repair mechanisms, making them particularly susceptible to treatments such as poly (ADP-ribose) polymerase (PARP) inhibitors [98, 99]. HRD and the resulting genomic instability also increase sensitivity to immunotherapy with ICIs [100]. NGS analysis revealed that 16% of CC patients have somatic pathogenic variants associated with HRD, highlighting the potential for evaluating the efficacy of HRD-targeted therapies and immunotherapy [101].

In a phase I study exploring sequential immunotherapy with ipilimumab following CRT for LACC, elevated levels of tumor-promoting cytokines (TNFα, IL-6, and IL-8) post-CRT were significantly associated with worse PFS [102]. The presence of CD4+ ICOS+ and CD4+ ICOS+ PD-1+ immune cell subsets was linked to significantly improved PFS. These findings highlight the potential role of these immune markers in predicting treatment outcomes for CC.

A potential biomarker for therapy intensification is circulating tumor HPV DNA (ctHPV DNA), which can be used to assess residual disease after CRT for LACC [103]. The persistence of ctHPV DNA was associated with worse outcomes, with patients who had detectable ctHPV DNA four to six weeks after treatment showing a two-year PFS of 15%, compared to 82% for those with undetectable disease (*P* < 001).

In summary, these potential predictive biomarkers provide valuable insights for personalizing treatment strategies, especially in guiding immunotherapy and other targeted treatments.

## Accessibility and global disparities

Despite the impressive strides in personalized therapy for CC, global access to biomarker testing and targeted treatments remains markedly uneven [104]. Low- and middle-income countries, which bear a disproportionately high burden of CC, often lack the infrastructure, specialized laboratories, and funding necessary to implement complex diagnostic assays (e.g., TMB, MSI testing) at scale. Although PD-L1 IHC is more readily available than some of the newer genomic assays (e.g., next-generation sequencing), its routine use can still be limited by staffing shortages, reagent costs, and quality assurance challenges. These obstacles are further compounded by the high costs of immunotherapies and novel agents such as antibody-drug conjugates, which frequently lie beyond the reach of most public health budgets.

Consequently, patients in these regions are often diagnosed at later stages and have fewer therapeutic options once diagnosed [105]. To bridge this gap, multi-stakeholder approaches involving government policy, industry-led tiered pricing, philanthropic initiatives, and international cooperation are needed. By subsidizing assay costs, establishing local testing networks, and negotiating reduced drug prices, it may be possible to extend the benefits of advanced biomarker-driven therapy to populations where CC incidence and mortality are most pronounced. Emphasizing capacity-building, education, and early detection strategies—such as HPV vaccination and screening—will also be critical in ensuring a more equitable global fight against CC. Continued international collaboration and commitment are essential to turning these proposals into reality.

## Conclusion

In conclusion, immunotherapy has significantly transformed CC treatment, substantially improving outcomes from second-line to first-line and locally advanced settings. However, to fully maximize its benefits in practice and improve tailored treatment strategies, the identification and implementation of reliable predictive biomarkers are essential. Ongoing basic and clinical research will further reveal the interplay between the immune system and CC, ultimately advancing more effective and personalized treatment approaches.
